# The Association between Multiple Sources of Information and Risk Perceptions of Tuberculosis, Ntcheu District, Malawi

**DOI:** 10.1371/journal.pone.0122998

**Published:** 2015-04-17

**Authors:** Robert Chizimba, Nicola Christofides, Tobias Chirwa, Isaac Singini, Chineme Ozumba, Simon Sikwese, Hastings T. Banda, Rhoda Banda, Henry Chimbali, Bagrey Ngwira, Alister Munthali, Peter Nyasulu

**Affiliations:** 1 Health Department, Save the Children International, Lilongwe, Malawi; 2 Department of Public Health, School of Health Sciences, Monash, South Africa; 3 School of Public Health, Faculty of Health Sciences, University of the Witwatersrand, Johannesburg, South Africa; 4 Johns Hopkins Research Project, College of Medicine, University of Malawi, Blantyre, Malawi; 5 Pakachere Institute of Health and Development Communication, Blantyre, Malawi; 6 Research for Equity and Community Health (REACH) Trust, Lilongwe, Malawi; 7 National Tuberculosis Control Program, Community Health Sciences Unit, Ministry of Health, Lilongwe, Malawi; 8 Health Promotion Services, Ministry of Health, Lilongwe, Malawi; 9 Department of Community Health, College of Medicine, University of Malawi, Blantyre, Malawi; 10 Centre for Social Research, University of Malawi, Zomba, Malawi; Fundació Institut d’Investigació en Ciències de la Salut Germans Trias i Pujol. Universitat Autònoma de Barcelona. SPAIN

## Abstract

**Background:**

Tuberculosis (TB) is one of the main causes of death in developing countries. Awareness and perception of risk of TB could influence early detection, diagnosis and care seeking at treatment centers. However, perceptions about TB are influenced by sources of information.

**Aim:**

This study aimed to determine the association between multiple sources of information, and perceptions of risk of TB among adults aged 18–49 years.

**Methods:**

A cross-sectional study was conducted in Ntcheu district in Malawi. A total of 121 adults were sampled in a three-stage simple random sampling technique. Data were collected using a structured questionnaire. Perceptions of risk were measured using specific statements that reflected common myths and misconceptions. Low risk perception implied a person having strong belief in myths and misconceptions about TB and high risk perception meant a person having no belief in myths or misconceptions and demonstrated understanding of the disease.

**Results:**

Females were more likely to have low risk perceptions about TB compared to males (67.7% vs. 32.5%, p = 0.01). The higher the household asset index the more likely an individual had higher risk perceptions about TB (p = 0.006). The perception of risk of TB was associated with sources of information (p = 0.03). Use of both interpersonal communication and mass media was 2.8 times more likely to be associated with increased perception of risk of TB (Odds Ratio [OR] = 2.8; 95% Confidence interva1[CI]: 3.1–15. 6; p = 0.01). After adjusting for sex and asset ownership, use of interpersonal communication and mass media were more likely to be associated with higher perception of risk of TB (OR, 2.0; 95% CI: 1.65–10.72; p = 0.003) compared with interpersonal communication only (OR 1.6, 95%; CI: 1.13–8.98, p = 0.027).

**Conclusion:**

The study found that there was association between multiple sources of information, and higher perceptions of risk of TB among adults aged 18–49 years.

## Introduction

Tuberculosis (TB) causes morbidity among millions of people each year and is ranked the second leading infectious cause of death worldwide, after the Human Immunodeficiency Virus (HIV). World Health of Organization (WHO) Global Tuberculosis Report 2013 [1] indicates that in 2012, an estimated 8.6 million people developed TB and 1.3 million people died. The majority of cases reported occurred in South-East Asia (29%), Africa (27%) and Western Pacific (19%) regions [1]. The TB incidence rates are high in Southern Africa with ≥1000 cases per 100 000 persons in countries such as South Africa and Swaziland. The proportion of TB cases co-infected with HIV-infection is highest in Sub Sahara Africa. An estimated 37% of TB cases are co-infected with HIV while in some parts of Southern Africa, more than 50% of TB cases are HIV co-infected [1]. TB infections in Sub-Saharan Africa mostly occur in the economically productive 15–49 years old age-group. In 2011 in Malawi, there were an estimated 20,000 new and relapse cases of TB and 1,900 deaths due to TB. The current national incidence of TB in the country is 147/100, 000 persons, with a HIV/TB co-infection rate of 60% [2].

Early diagnosis of TB among suspected individuals could prevent occurrence of new infections and eventually reduce TB deaths [3,4]. However, early TB diagnosis is hampered by perception of low risk as well as other factors such as age of individuals, level of education, household asset index, gender differences and previous history of TB diagnosis [5–8]. In a study carried out by Dias et al in Brazil among TB patients who completed their treatment, perception of low risk of TB was associated with negative attitudes about TB, lack of recognition of signs and symptoms of TB, high stigma and belief in myths among community members, as well as misconception that TB is incurable. These factors affect early care seeking behaviour for TB, consequently hampering early prevention and control of the disease [9]. Poor perceptions of TB disease, often delays care seeking until occurrence of severe pulmonary symptoms such as shortness of breath; coughing out sputum with blood and weight loss. However, individuals diagnosed with TB tend to show higher knowledge as well as less belief in myths regarding TB when compared with non-TB patients [5–8].

Source of information, has been shown to influence individuals’ perceptions about TB disease. Channels of communication identified include: personal experience, mass media (radio, television, newspapers), interpersonal channels such as health workers, personal experience, previously treated for TB, family and friends, teachers and community members, print media (leaflets and booklets) and lectures [5, 10–13] Dias et al., [5] in their study showed that people who obtained information through interpersonal communication were more likely to seek early TB diagnosis and complete treatment course compared with those who obtained information through other means. However, Waisbord [14] found that people who obtained information through interpersonal communication alone showed limitations in overcoming barriers to TB prevention and control. Furthermore, Hoa et al., [13] found that although mass media created general awareness about TB, it was not effective in reducing peoples’ perception of risk of TB. In a study carried out in Nigeria by Ugochukwu et al., [10], the need for a combination of communication formats and approaches to achieve effective outcome in behavioural change regarding TB disease was supported further.

The aforementioned literature emphasize the need for use of multiple sources of information, however recent TB research in Malawi has focused mainly on the bio-medical component [7, 8] while earlier TB research focused on the incidence rates of TB and the local perceptions of the disease [15–17]. No known study has been done in Malawi, to determine the association between multiple sources of information and perception of risk of TB, as well as knowledge among adults aged 18–49 years, in a rural district setting in Malawi.

## Methods

### Study design

This was analytical cross-sectional study that focused on the association between multiple sources of information and risk perceptions of TB.

### Study setting and population

The study was conducted in January 2012 in Ntcheu district with a total population of 474,464. Of these, 226,567 were males and 247,897 females [18]. A total of 227,134 people were aged above 18 years of which 103,440 were males and 123,694 were females [18]. The district is served by nine Traditional Authorities (TAs) namely: Phambala, Mpando, Kwataine, Makwangwala, Champiti, Njolomole, Chakhumbira, Goodson Ganya and Masasa. The district has one hospital known as Ntcheu District Hospital, and 36 Health Centres. Ntcheu district has the highest TB incidence in Malawi estimated at over 57 per 100,000 persons [19]. In 2010 alone, Ntcheu district registered 525 TB cases. The study population included adults aged 18–49 years since high morbidity and mortality related to TB in low income countries predominantly occur in this age-group [20]. The district has three main tribes, namely Ngoni, Chewa and Yao. Of the three tribes Ngoni is the predominant tribe and Chichewa is the main language.

### Sampling and study sample-size

To assess TB knowledge base, the communities were conveniently chosen based on their similarity in population dynamics, number of health facilities, socio-economic status, poverty levels, literacy rate, culture of the people, TB disease burden and challenges in tuberculosis control. The study used a three-stage sampling technique. The first stage involved simple random sampling of two Traditional Authorities (TAs) from the nine TAs in the district using computer generated random numbers. Based on this process, two TAs namely Chakhumbira and Makwangwala were selected. Stage two involved random selection of 10 villages each from TA Chakhumbira and TA Makwangwala. Using the 2008 Malawi Population and Housing Census maps obtained from the National Statistical Office in Zomba, all existing demarcated enumeration areas (EAs) in TA Chakhumbira were used. A total of 49 villages from 23 EAs in Chakhumbira formed a sampling frame. The same process was repeated for TA Makwangwala where a total of 199 villages from 79 EAs formed a sampling frame. A total of six households in each of the 20 villages were selected. A household in this survey was defined as people living together and eating from the same pot. Names of households in each village were solicited from the village head or his/her representative and were arranged alphabetically. The first household was randomly sampled and subsequent households were systematically selected by going to the third household on the right hand side of the main entrance of the previous household until the required sample size of six households in each village was reached. Convenient sampling was used to select a participant per household, whereby, the first person found at a household and who fell within the age-group 18–49 years was interviewed. At any household, no sampled person refused to participate in the study. A total of 121 adults aged 18 to 49 years were interviewed.

The required sample size was determined using the following formula for prevalence surveys:

$$n=\frac{t^{2}\times p\left(1-p\right)}{m^{2}}+r$$

Where

**n** = required sample size

**t** = confidence level at 95% (standard value of 1.96).**p** = estimated proportion of stigma in Malawi set at 50% (therefore p = 0.5).

**m** = margin of error set at 10% and **r** = non response rate set at 20%. The non-response rate was set at 20% as it was presumed that people would not be willing to participate in this TB study as the disease is closely associated with HIV/ AIDS stigma.

### Data collection procedures

Experienced interviewers collected data through face-to-face interviews in the local language (Chichewa) from participants in their homes using a structured questionnaire. The questionnaire included only closed questions. The enumerators were trained by the principal investigator to understand the study objectives, TB concepts, ethical issues and standard operating procedures regarding data collection. After training, the entire study process and tools were piloted in TA Nsomba in Blantyre district which is about 120 kilometres away from Ntcheu. The piloting exercise included: sampling of respondents at household level, conducting face-to-face interviews, feasibility of completing interviews, timing for questionnaire completion and storage of completed questionnaires. Data collection in the field site was done in January 2012.

### Variable measurements

To measure socio-economic characteristics of the respondents these parameters were used: a single item on average monthly household income on a scale of 1–4; a single item that measured access to water on a scale 1–4, a single item on sanitation on a scale 1–4 and three items that measured quality of housing on a scale 1–3. An additional measure of socio-economic status was a list of household assets (radio, television, cell phone, chairs etc.). These items were then re-coded to create two scores. The continuous variables such as socio-economic status (SES) and ownership of household asset score were dichotomized into higher and lower SES. On the measurement of risk perceptions of TB, the participants indicated their agreement with the item on a four-point Likert scale. Perceptions of risk of TB was measured using five parameters as follows: beliefs that one cannot get TB because the person looks healthy in physical appearance; one can get the disease only if he/she is HIV positive; belief that one should not worry about TB as it is easily treated; TB is common and any person can acquire the infection. These items were then added to create a score for each sub-scale. The internal consistency of the sub-scales was assessed using a Cronbach’s method. The distribution of the scale was reviewed for normal distribution. If there was normal distribution, the continuous variables were dichotomized based on a median split into a higher or lower risk perceptions or higher and lower knowledge. Sources of media were categorized as interpersonal (face-to face) and mass media (use of radio, television or print materials that would reach more people within a period). A checklist was used each day to assess how well the enumerators administered the questionnaires as well as the length of time taken for each interview to be completed. Questionnaires were also checked in the field at the end of each day to assess completeness and accuracy of records. A daily interview form was used where information on the number of interviews completed each day per enumerator was recorded.

### Ethical consideration

The study was approved by the Human Research Ethics Committee of the University of the Witwatersrand, Johannesburg and the Health Sciences Research Committee, Ministry of Health, Lilongwe, Malawi. All participants gave a written informed consent before participating in the study.

### Data analysis

Data were entered into a Statistical Package for Social Scientists (SPSS) database and consistency and range checks were performed in Excel spread sheet. Further data cleaning procedures were conducted to assess inconsistencies and data errors. Statistical analysis was done using STATA version 12.0. A Chi-Square test of independence used to determine the association between exposure factors and TB risk perception. Fisher’s exact test for small sample sizes was used to ascertain the statistical significance of observed differences at every level. Multiple regression analysis of sources of information and risk perceptions was done to determine the association between multiple sources of information and risk perceptions of TB. Odds ratios were used as measure of effect.

## Results

### Socio-demographic characteristics of the study participants

A total of 121 participants were interviewed, of these 53.7% were males, median age 28 years (inter-quartile range = 15.5 years). Among the participants 25.6% had no formal education and 20.6% had completed secondary school level. Sixty-three percent (63%) were below the low social economic status scale (mean = 10.2, SD = 1.7). Radio (75.2%) was the most frequently reported media channel through which people obtained information about TB, followed by health centres/clinics (68.3%) and newspapers (5.6%) (Table 1).

**Table 1:** Distribution of socio-demographic characteristics of the participants.

Socio-demographic characteristics	Frequency	Percentage
**Age group (Age n = 116)**		
18–20	25	21.6
21–25	21	18.1
26–30	20	17.2
31–35	19	16.4
>35	31	26.7
**Sex n = 121**		
Males	65	53.7
Females	56	46.3
**Education n = 121**		
None	31	25.6
Standard/Grade 5	28	23.1
Standard/Grade 8	37	30.6
Secondary	25	20.7
**Average monthly household income n = 107**		
Under US$10/month	45	42.1
US$11-US$16.66/month	21	19.6
US$17.66-US$33.33	17	15.9
>US$33.33	24	22.4
**Ownership of Household assets n = 121**		
Low	80	66.1
High	41	33.9
**Number of people in a household n = 121**		
1–2	15	12.4
2–4	48	39.6
5–7	51	42.1
8+	7	5.9
**Ever been diagnosed with tuberculosis n = 119**		
Yes	36	30.3
No	83	69.7
**Sources of information, a person could list more than one source n = 273**		
Health Centre/Clinic	82	30.0
Relative of the participants	30	11.0
Village headman	15	5.5
Community-based organisation	33	12.1
Radio	91	33.3
Television	15	5.5
Newspapers	7	2.6

### Distribution of perception of risk of TB by socio-demographic characteristics of the participants and sources of information

Perception of risk of TB was assessed and scores on high or low perception of risk were aggregated. There was significant difference in perception of risk of TB between males and females as well as between those with low and high household asset index. Females were more likely to have low risk perceptions about TB compared with males *(p = 0*.*01)*. A higher perception of risk of TB was associated with higher household asset index *(p = 0*.*006)*. In addition, there was higher perception of risk of TB associated with combined use of different sources of information i.e. use of both mass media and interpersonal communication *(p = 0*.*03)* (Table 2).

**Table 2:** Comparison of socio-demographic characteristic of participants by risk perception of TB.

Socio-demographic characteristics	High TB risk perceptions N (%)	Low TB risk perceptions N (%)	P-value *
**Age group n = 116**			
18–20	16(20.3)	9(24.3)	0.96
21–25	15(19.0)	6(16.2)	
26–30	14(17.7)	6(16.2)	
31–35	12(15.2)	7(18.9)	
>35	22(27.9)	9(24.3)	
**Sex n = 120**			
Males	51(63.8)	13(32.5)	0.01
Females	29(36.2)	27(67.5)	
**Education n = 120**			
None	15(18.8)	15(37.5)	0.11
Standard/Grade 5	19(23.8)	9(22.5)	
Standard/Grade 8	29(36.3)	8(20.0)	
Secondary	17(21.3)	8(20.0)	
**Average monthly household income n = 106**			
Under US$10/month	26(37.1)	18(50.0)	P = 0.35
US$11-US$16.66/month	17(24.3)	4(11.1)	
US$17.66-US$33.33	12(17.1)	5(13.9)	
>US$33.33	15(21.4)	9(25.0)	
**Ownership of Household assets n = 120**			
Low	46(57.5)	33(82.5)	P = 0.006
High	34(42.5)	7(17.5)	
**Number of people in a household n = 120**			
1–2	9(11.3)	5(12.5)	P = 0.66
3–4	30(37.5)	18(45.0)	
≥5	41(51.2)	17(42.5)	
**Ever been diagnosed with tuberculosis n = 119**			
Yes	51(64.6)	32(80.0)	0.80
No	28(35.4)	8(20.0)	
**Sources of information a person could list more than one source n = 119**			
Mass media only	8(10.0)	9(23.1)	0.03
IPC**	14(17.5)	11(28.2)	
Both Mass Media & IPC**	58 (72.5)	19(48.7)	

Note: *p< 0.05 is significant;

**Inter-Personal Communication

### Factors associated with perception of risk of tuberculosis

In multiple logistic regression analysis, factors independently associated with perception of risk of TB were sources of information and asset ownership. Individuals who received information about TB through both interpersonal and mass media were nearly three times more likely to demonstrate low perception of risk of TB *(OR = 2*.*8; p = 0*.*01)* compared with interpersonal communication only. After adjusting for sex, use of both sources of information (i.e. interpersonal and mass media) showed twice the odds of perception of risk of TB compared with interpersonal communication only (OR = 2.0, *p = 0*.*003)* and 1.6 times the odds of perception of risk of TB after adjusting for household asset ownership (OR 1.6, *p = 0*.*02*). (Table 3)

**Table 3:** Factors associated with risk perceptions of tuberculosis: Sources of information while adjusting for sex, ownership of assets and ever been diagnosed with TB.

Socio-demographic characteristics and sources of information	Adjusted OR	(95% CI)	p-Value*
IPC	2.6	(0.97–15.35)	0.097
Both mass media and IPC**	2.8	(1.31–15.56)	0.017
Sex	2.0	(1.65–10.72)	0.003
Ownership of assets	1.6	(1.13–8.98)	0.027
Ever been diagnosed with TB	1.3	(0.95–7.04)	0.062

Note: *p< 0.05 is significant

Note: **Inter-personal Communication

## Discussion

Our present study assessed risk perceptions of TB relating to the use of interpersonal communication versus combined source of information, among other factors. This study has shown that sources of information and income status are key in determining perception of risk of TB in this rural population. Individuals who are exposed to mass media and interpersonal communication have increased perception of risk of TB when compared with individuals exposed to only interpersonal communication. Similarly, individuals with better income seem to have higher perception of risk of TB than those who are poorer. This could be due to the possibility that those who have higher income may also afford to own mass media technology such as TV and radio. This suggests that use of interpersonal and mass media communication channels together might have a greater impact in apprehension of community members’ perception of risk of TB infection. An effective health promotion model to enhance early TB diagnosis should therefore target use of dual mode of communication. In addition the study has shown that gender differences exist in perception of risk of TB with males having a higher perception of risk of TB than females. This is despite the fact that females have greater contact with the health care services through antenatal care and under-five immunisation services, which are potential sources of information. According to the Malawian National Tuberculosis Control Programme manual [21], “health workers attending to patients at outpatient departments in every health delivery setting should be very familiar with TB as a disease as well as common TB diagnostic procedures. Health workers should ensure that frequent health education sessions are conducted in all OPD departments by the District TB Officer, focal clinician, focal nurse or the Health Education Officer” [21]. If this protocol which is part of the TB control programme was strictly followed, women attending antenatal care and under-five immunisation services should be knowledgeable about causes and symptoms of TB and associated interventions. This level of knowledge is expected to reflect in women’s perception of risk of TB disease. Therefore, this might be an indication that there is limited integration of information regarding maternal and child health services and TB in Ntcheu district, in Malawi.

The results of this study support other findings of the relationship between sources of information and risk perceptions about TB. In their study, Dias et al., [5] found that people who got information through multiple communication channels were more likely to have higher perception of risk (i.e. recognized signs and symptoms of TB, sought early diagnosis and completed treatment) than those who got the information from interpersonal or mass media only. Other studies have also shown that ownership of household assets is associated with perception of risk of TB disease; the more the household assets an individual has the higher the perception of risk of TB disease [5–8, 10].

These findings underscore the fact that an effective health promotion intervention should target a combined approach in the dissemination of health promotion and awareness information about TB to improve early detection, diagnosis and access to treatment of TB among individuals in the rural communities. The results of this study are, therefore, in keeping with previous reports that suggest that an effective health promotion program should use communication methods that should be delivered to the communities through use of multiple sources of information as opposed to a single source of information [13, 22].

The findings of this study have implications on the implementation of the health education and awareness model, in Malawi. The model is a health promotion intervention based on a combination of social and behaviour change communication processes that uses multiple channels. The Ministry of Health through the National TB Control Programme [21] has been implementing several TB control measures, however, much of these activities have focused on the bio-medical platform and have not addressed demographic and socio-economic factors which are crucial in preventing and controlling TB in rural communities through early reporting and enhanced diagnosis of symptomatic disease. The results of this study will therefore inform development of an effective health promotion intervention that should target essential socio-economic risk factors that propagates TB disease through an exposition of multiple sources of information about TB rather than the traditional single source of information.

### Limitations

There is possibility of sampling bias playing a role in the findings of this study due to the fact that field work was conducted during the rainy season when most of the older people were busy in the fields cultivating. This may have lead to an over-representation of individuals aged 18–28 years in the sample. TB diagnosis was self-reported and this could have resulted in under-reporting as TB is highly stigmatised in this area. Under-reporting would have biased the results towards the null hypothesis. Internal consistencies for some scales used to assess knowledge level, risk perceptions and socio-economic status were not optimal. The study did not measure the relationship between the perception of risks of TB and mass media as an information source only therefore mass media could have confounded the observed association.

### Conclusion

The study has shown a relationship between use of multiple sources of information about TB and increased perceptions of risk of TB among people in Ntcheu district. These results will inform development of a comprehensive health promotion programme that will implement use of multiple sources of information to increase perception of risk of TB thereby enhancing early TB diagnosis and access to TB treatment.

## Supporting Information

S1 Dataset (XLSX)
